# Role of in vivo dosimetry with radiochromic films for dose verification during cutaneous radiation therapy

**DOI:** 10.1186/s13014-014-0325-0

**Published:** 2015-01-10

**Authors:** Hong-Wei Liu, James Gräfe, Rao Khan, Ivo Olivotto, J Eduardo Villarreal Barajas

**Affiliations:** Central Alberta Cancer Center, 3942-50a Ave, Red Deer, AB T4N 6R2 Canada; Department of Oncology, University of Alberta, Edmonton, AB Canada; Tom Baker Cancer Center, Calgary, AB Canada; Department of Oncology, University of Calgary, Calgary, AB Canada

**Keywords:** GAFCHROMIC EBT3™ film, Extended source surface distance, Orthovoltage, Inverse square law, Quality assurance

## Abstract

**Purpose:**

To evaluate the role of in vivo dosimetry with radiochromic films for dose verification in cutaneous radiation therapy (RT).

**Methods:**

Five patients with 8 cutaneous or sub-cutaneous malignancies of the face, neck, trunk and extremity receiving RT were included. Orthovoltage, megavoltage photon therapies were applied based on anatomic location. The delivered dose for each target was measured with GAFCHROMIC EBT3TM film. The differences between the prescribed and measured doses in each target were analyzed based on the RT characteristics, target location and custom patient set up. The accuracy of EBT3TM film measurement was verified by measurements in a solid water phantom.

**Results:**

The mean measured dose was -3.2% (-9.6% to +2.3%, P=0.86) lower than prescribed over 23 measurements. A wide range of under dose was detected in orthovoltage therapy when a gap existed between skin and a closed-ended applicator surface. The magnitude of the under dosage was correlated with the degree of the gap (P=0.01). The phantom study confirmed the accuracy of GAFCHROMIC EBT3TM film measurement and found that the low measured dose in orthovoltage therapy was caused by the deviation from the inverse square law (ISL) of the beam output at extended source surface distance (SSD) for closed-ended applicators.

**Conclusions:**

A significantly low delivered dose for extended SSD orthovoltage therapy was demonstrated during cutaneous RT. The dose fall-off with distance is not completely compensated by the ISL standoff correction for orthovoltage therapy. GAFCHROMIC EBT3™ film is a useful and accurate tool for quality assurance of patients receiving a curative intended cutaneous RT.

## Introduction

Primary cutaneous malignancies mainly include basal cell carcinoma (BBC) and squamous cell carcinoma (SCC). BCC is usually slow growing, rarely metastasizes and may be cured with effective local therapy including surgical resection, radiation therapy (RT) or both [1-3]. RT for primary BCC has achieved local control rates of 80-100% [4-6]. RT is also commonly used for symptom relief in metastatic cutaneous malignancies.

Orthovoltage X-rays or megavoltage (MV) energy electrons are the main cutaneous radiotherapy tools [3,7,8]. MV photon therapy is used for large, subcutaneous targets with deep tissue involvement. Commonly, cutaneous RT is delivered with an appositional field using a clinical set up and without 3D volumetric dose calculations. A percent depth dose curve is used to calculate target dose coverage based on estimated tumor depth. Compared to MV electron and photon treatments, orthovoltage X-ray treatment units are relatively simple with set-up reproducibility guided by the use of an applicator (often called a “cone”) directly onto the target surface. Orthovoltage beams deposit the maximal dose at the skin surface and have a smaller penumbra than MV electron and photon. A custom lead (Pb) cut-out can be used to define irregularly shaped fields. However, sometimes there is a gap (standoff) between the target surface and the applicator. In such circumstances a standoff correction using the inverse square law (ISL), is used to account for the impact of dose fall-off due to distance from the applicator end.

Accurate dose delivery is important to achieve and report control rates during curative-intent cancer therapy. It is possible that delivered dose may be different from the estimated dose due to unanticipated or under-appreciated skin gaps, tissue folds, or set-up and positioning variation.

GAFCHROMIC EBT3™ film has an active layer of radiation-sensitive organic microcrystal monomers on thin polyester located between two identical polyester layers. The film is near tissue equivalent. EBT3™ film is highly sensitive to ionizing radiation. These features make it potentially suitable as a pragmatic clinical dosimeter. The aim of the current study was to examine the clinical value of GAFCHROMIC EBT3™ film as an *in vivo* dosimeter for quality assurance during cutaneous radiotherapy. The manufacturer of GAFCHROMIC EBT3™ film did not provide any funding or have any input into the study design, conduct, analysis or reporting.

## Materials and methods

### Patients and targets

Five consecutive patients including one male and four females received RT to 8 cutaneous or sub-cutaneous lesions of the face, neck, trunk and extremity by a single radiation oncologist. The median age was 86 years (range 49–91 years). Skin lesions included primary BCC, SCC and metastatic melanoma. This investigation was considered a quality assurance study by the institutional research ethics board but individual consent was obtained from each patient prior to the film dosimetry measurements.

### Radiation therapy

The RT dose was measured by placing film on the target surface for patients who received orthovoltage X-ray therapy. Additionally, one patient with MV photon therapy was included for comparison. Patients with lesions of the face, neck or chest wall skin were treated with orthovoltage photons using an Xstrahl-300 X-ray therapy unit (Xstrahl Ltd., Camberley, UK) with treatment energies of 150 and 200 kVp and half-value layers of 6 mm Al and 1 mm of Cu, respectively. Treatment was delivered with either open-ended circular applicators (diameters of 1.5 or 3 cm) at 30 cm focus to skin surface distance (FSD) for small lesions or closed-ended square applicators at 50 cm FSD for larger lesions. Custom Pb cut-outs were manufactured for large irregularly shaped lesions treated with orthovoltage. One patient with a SCC of the left wrist had a positive margin after amputation and received adjuvant RT with a single 6 × 7 cm^2^ field using 6 MV photons. A mould of the patient’s hand and wrist was made out of Polyethylene Terephthalate Glycol. The mould was mounted to a 10 × 22 × 30 cm^3^ plastic box filled with LiquiBlock™ to provide sufficient backscattering and build-up for a 6 MV photon beam LiquiBlock™ is a non-toxic absorbent polymer salt that when mixed with water forms a jelly-like substance used for bolus. The jelly was distributed so that 1–1.5 cm of build-up covered the target area. A 101 cm source to skin distance (SSD) was set to the skin surface. Table 1 presents detailed information about the RT delivery parameters.

**Table 1:** **Radiation therapy delivery parameters for the eight clinical lesions**

**Target**	**Pathology**	**Field size**	**Applicator size**	**SSD**	**Skin bolus**	**Energy**
**Location**		**(cm)**	**(cm)**	**(cm)**	**(cm)**	
Pre auricular	BCC	3.0	3 circle	30	0	150 kVp
Medial canthus	BCC	1.5	1.5 circle	30	0	200 kVp
Nose tip	BCC	1.5 × 1.5	1.5 circle	30	0	150 kVp
Medial neck	BCC	6 × 6	10 × 10	50	0	200 kVp
Chest wall skin	MM	3 × 3	3 circle	30	0	200 kVp
Chest wall skin	MM	3 × 3	3 circle	30	0	200 kVp
Chest wall skin	MM	5 × 6	6 × 6	50	0	200 kVp
Left wrist	SCC	7 × 6	-	101	1	6 MV

SCC = Squamous cell carcinoma; BCC = Basal Cell Carcinoma; MM = Metastatic melanoma; kVp = orthovoltage energy; MV = Mega Volts.

### RT dose calculation and measurement

RT doses were prescribed, and film measurements were taken at the skin surface. Monitor units (MUs) for orthovoltage treatment were calculated based on the prescribed daily dose modified by a back scatter factor (BSF), ISL if there was standoff from the skin surface to the applicator, and the machine output factor. The ISL correction factor or standoff correction (SOF) factor was defined as: I _ISL_ = [FSD_N_/(FSD_N_ + S)]^2^ = SOF, where FSD_N_ is the nominal FSD (30 cm for open-ended and 50 cm for closed-ended applicators), and S is the amount of standoff in cm. MUs for the MV photon treatment were calculated using standard tissue-maximum ratios.

The delivered dose for each target was measured with GAFCHROMIC EBT3™ film (International Specialty Products, Wayne, NJ, USA) for a minimum of three fractions per lesion (except one lesion had two). EBT3™ film was placed on the skin surface. The goal was to measure a point dose at the centre of the lesion. For each case, the film was cut to conform to the lesion size and location. If there was an irregular surface, the film was cut small enough to be placed directly on the surface at the center of the field. The smallest film was at least 1 × 1 cm^2^ and the largest film was 6 × 6 cm^2^. Differences between prescribed and measured doses were calculated as: (Dose_M_ –Dose_p_)/Dose_p_ × 100%, where Dose_p,_ and Dose_M_ were the planned and measured doses, respectively.

A single batch (Lot# A05151203) of EBT3™ film was used for all measurements because the film response and calibration curves can be batch specific [9]. Films were stored in a light-proof container to minimize UV contamination. The films were scanned with an Epson Expression 10000XL flat-bed scanner (Epson America, Inc. Long Beach, CA, model XL 10000). Based on scanner uniformity (pixel value variation), dose calibration curve fitting, and measurement reproducibility, it was estimated that the film dosimetry protocol accuracy varied by 3-5% for doses from 800 cGy down to 200 cGy. Therefore, measured systematic dose deviations greater than 5% warranted further investigation.

### EBT3 film calibration, phantom measurements and ion chamber dose verification

The calibration films were cut into 4 × 4 cm^2^ pieces. Due to the energy dependence of EBT3 films from the kilovoltage range to the MV range [10], separate calibration curves were calibrated for 150 kVp and 200 kVp beams, and a ^60^Co unit (Theratron 780-C; Best Theratronics, Ottawa, ON, Canada) was used to calibration for the MV beam. The Xtrahl orthovoltage treatment beam was calibrated in absolute dose following the American Association of Physicists in Medicine (AAPM) TG61 guideline [11], and the ^60^Co unit was calibrated following the AAPM TG51 guideline [12]. A minimum of two films were used for each dose level of the calibration curve, and the films are scanned in RGB color mode using only the red color channel. This produced a calibration curve of dose in cGy against net optical density. Film handling and scanning protocol followed the procedure described previously [10].

**Figure 1:**
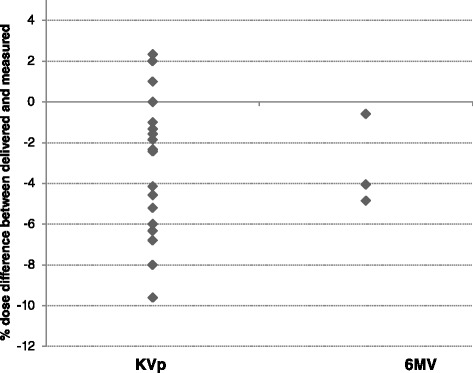
**Comparison of the prescribed dose with the EBT3™ film measured dose for MV photon, and orthovoltage X-ray radiation therapy (N = 23).**

**Figure 2:**
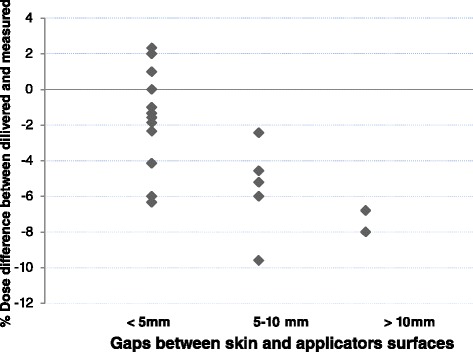
**Correlation of the dose difference measured with EBT3™ film with air gap (<5 mm, 4 sites; 5-10 mm 2 sites; >10 mm, 1 site) during orthovoltage radiation therapy (P = 0.01).**

A systematic difference between measured and prescribed dose was noticed for patients treated on orthovoltage in whom a standoff correction had been calculated (Figures 1 and 2). To investigate the dose deviations from the ISL estimates, the EBT3 film was placed on a solid water phantom and the number of MUs required to deliver a dose of 700 cGy with a 10 × 10 cm^2^ square, closed-ended applicator with no standoff (i.e. 0 cm) was delivered at 0, 1, 2, 3, and 5 cm standoff distances. The same experiment was repeated with a Markus plane parallel ionization chamber (PTW Frieburg, Germany; model N23343) in air for verification. A detailed description of the ion chamber measurements has been reported previously [13]. To directly compare the film results from the phantom surface with the in air ion chamber measurements, the field size dependent BSF from AAPM TG61 were factored out to account for phantom backscatter and increase in field size on the water phantom due to beam divergence with increasing standoff [11].

### Statistical analysis

The mean differences between film-measured and prescribed doses were compared using a two-tail Student’s paired t-test with a cut-off p-value < 0.05 to indicate statistical significance. ANOVA was used to analyze the differences between group means and their associated variation.

## Results

We evaluated over 23 measurements for 8 lesions. On average the film measurements were 3.2% lower (−9.6% to +2.3%, P = 0.86) than the prescribed doses (Figure 1). For the 6 MV photon case, the measured dose was 3.2% lower than prescribed (range, −4.9 to −0.6%). Doses measured during orthovoltage treatments showed wide deviations from the prescribed doses. A subgroup classification based on the ranges of gaps between the skin surface and the end of the applicator cone (N = 20 observations) is presented in Figure 2. Under dosage of more than 5% was detected when there was a gap of > 5 mm between the skin and a closed-ended applicator surface. Using ANOVA the degree of the under dosage was well-correlated with the degree of the gap distance (P = 0.01).

**Figure 3:**
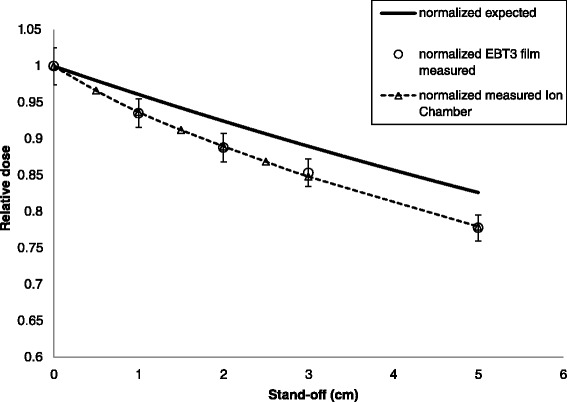
**Relative dose fall-off with standoff using a 10 × 10 cm** ^**2**^ **closed-ended applicator measured with EBT3 film placed on a solid water phantom and compared to the expected fall-off based on the inverse square law.** The BSF was factored out of the film measurements for direct comparison with the in air ion chamber measurements and the expected fall-off. Excellent agreement is demonstrated between ion chamber and film.

The observed correlation between standoff and measured under dosage was investigated using water phantom measurements. After factoring out the impact of back scatter, the film measurements compared those measured with an ion chamber were well within experimental uncertainty. The dose fall-off with gap distance measured by the EBT3™ film was greater than predicted by the ISL standoff correction as shown in Figure 3. The physical set-up details and investigations for the ion chamber measurements have been reported in a separate publication [13].

## Discussion

In the current study, film-measured dose during orthovoltage therapy displayed large deviations from calculated doses in particular, when there was standoff of 1 cm or greater. The Xstrahl-300 orthovoltage unit was commissioned for clinical beams of 100, 150, and 200 kVp using a range treatment delivery device from 1.5 cm circles to 20 × 20 cm^2^ open-ended or closed-ended applicators. The clinical decision to use a particular applicator is based on tumor size, lesion depth and location. When an air gap exists between the skin surface and the end of the applicator, an ISL correction is used to adjust the number of MUs delivered to compensate for beam divergence. When the standoff gap was larger than 1 cm, the dose measured with the EBT3™ film was significantly lower than the prescribed dose and the extent of under dosage was directly proportional to the size of the air gap between the skin surface and the end of the treatment unit applicator (Figure 2). Applying the traditional ISL correction for the standoff did not fully compensate for the actual dose drop.

**Figure 4:**
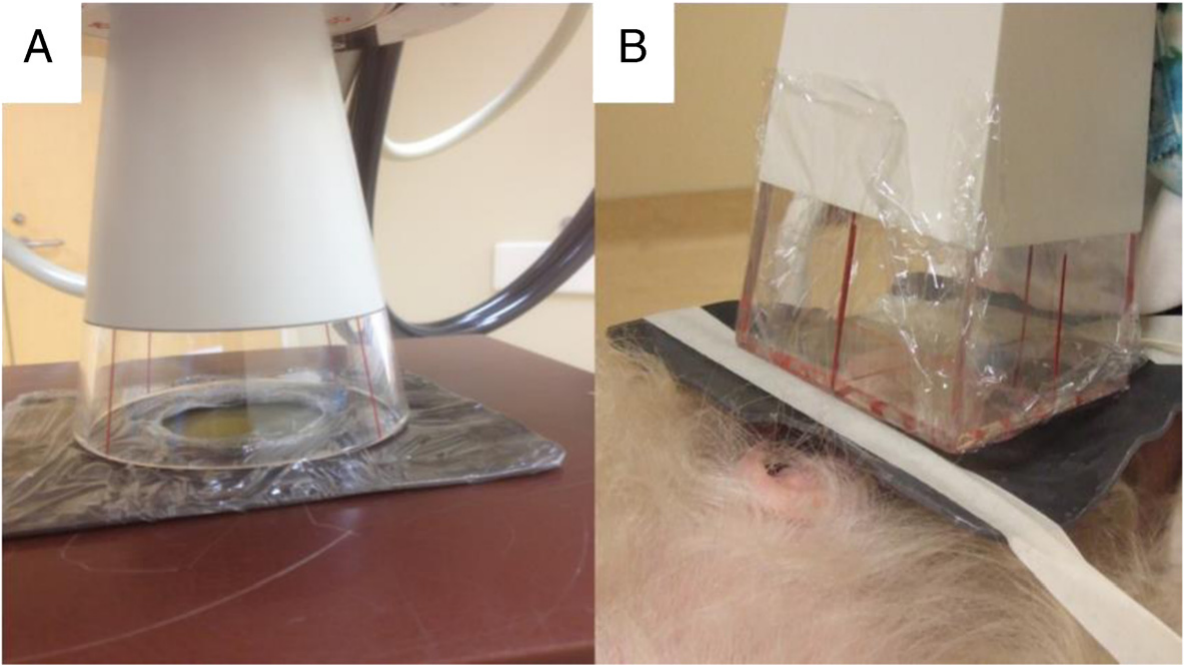
**Illustration of the gap between skin and cone surface during an orthovoltage delivery. A**. Open-ended applicator with no gap vs. **B**. Closed-ended applicator with a gap in the clinical set up.

A 1–2 cm air gap is most likely to occur when a closed-ended applicator is applied to an irregular surface (Figure 4). For larger tumors using a 50 cm FSD and a closed-ended cone, parts of the field may experience gaps of more than 1 cm. The convergence of measured doses using EBT3 film on water phantoms compared to in air ion chamber measurements confirms that the relative dose decline is a function of incomplete standoff correction when using an ISL correction alone. Several phantom studies have confirmed that EBT3™ film can be used for quality assurance in a variety of radiotherapy applications, including at orthovoltage energies [10,14,15] but this is the first to report the discrepancy between film-measured doses and ISL adjusted, prescribed doses for patients with air gaps of 1 cm or greater.

Previously [13], we quantified the deviation from the ISL of closed-ended applicators using an orthovoltage treatment unit, as has been reported by others [16,17]. The mechanism of the under dosage at extended SSD is attributed to the endplate of closed-ended applicators. The 4 mm acrylic end-plates of the Xstrahl-300 clinical applicators act as an additional X-ray scatter source and effectively reduce the effective FSD of the clinical orthovoltage beam. This causes a more rapid dose fall-off than predicted by a basic ISL correction. The independent phantom film study and ion chamber measurements confirmed that the lower doses observed in the patient measurements were reproducible and quantifiable [13]. The divergence between calculated and measured doses became clinically significant when there was a large gap between the skin surface and the closed-ended cone even after an ISL correction was made because the ISL correction does not account for end-plate scattering [13]. Using only the inverse square law to account for a gap more than 1 cm might result in an under estimated dose of 5% or more.

The film measurements from 6 MV photon revealed non-significant dose difference between estimated and delivered. Therefore, no more case studies were further performed. This was in line with our initial study objectives of any systematic uncertainty greater than 5% warranted further investigation. The concordance of doses during the MV photon treatment in this study could be considered an additional verification of the systematic variation noted between the film-measured and prescribed doses when using orthovoltage energy with closed end applicators with increasing applicator to skin gaps.

Only small dose variations were observed between fractions. This is attributed to variations in patient set-up, reproducibility of skin lesion positions and uncertainties in placement of the film dosimeter between fractions. The current results suggest that a simple, *in vivo* dosimeter should be part of treatment planning quality assurance during curative intent cutaneous RT. At the very least it should be used for initial treatments on a new orthovoltage unit, and when there is measurable standoff between the applicator end plate and the skin surface.

Cutaneous cancers occurring on irregular skin surfaces such as the face, ear or neck are commonly seen in the clinic. Orthovoltage RT provides a simple, non-invasive alternative to surgical excision and can achieve high rates of complete response and local control. For small lesions encompassed in open-ended cones without air gaps, calculated doses are reliable estimates of the delivered dose. For larger lesions and lesions located on irregular surfaces treated with closed-ended applicators, a 1 cm or larger gap requires a larger correction than estimated using the ISL correction. For such cases, EBT3™ film-based *in vivo* dosimetry should be used as a quality assurance tool that can help to assess dose delivery. Minimization of large skin gaps is crucial to avoid unacceptable under dosage.

## Conclusion

EBT3™ film-based *in vivo* dosimetry demonstrated that delivered doses were significantly lower than calculated doses for extended SSD orthovoltage techniques using closed-ended applicators. Dose fall-off due to air-gaps as frequently occur when treating irregular surfaces were not completely compensated by the inverse-square law corrections. This is commonly neglected in clinical practice. *In vivo* film measurements can be used to improve quality assurance during curative intent cutaneous radiotherapy.
